# Profiling of relaxin and its receptor proteins in boar reproductive tissues and spermatozoa

**DOI:** 10.1186/s12958-015-0043-y

**Published:** 2015-05-20

**Authors:** Jean M Feugang, Jonathan M Greene, Hector L Sanchez-Rodríguez, John V Stokes, Mark A Crenshaw, Scott T Willard, Peter L Ryan

**Affiliations:** Department of Animal and Dairy Sciences, Facility for Organismal and Cellular Imaging (FOCI), Mississippi State University, Mississippi State, MS 39762 USA; Department of Pathobiology & Population Medicine, Mississippi State University, Mississippi State, MS 39762 USA; Department of Basic Sciences, Flow Cytometry facility core, College of Veterinary Medicine, Mississippi State University, Mississippi State, MS 39762 USA; Department of Biochemistry and Molecular Biology & Entomology and Plant Pathology, Mississippi State University, Mississippi State, MS 39762 USA; Department of Pathobiological Sciences, Robert P. Hanson Biomedical Sciences Laboratories, University of Wisconsin, Madison, WI 53706 USA; Department of Animal Science, Mayaguez Campus, University of Puerto Rico, Mayaguez, Puerto Rico

**Keywords:** Fertility, Immunofluorescence, Leydig cells, Pig, Relaxin, Semen, Seminal plasma, Sertoli cells, Sperm motility

## Abstract

**Background:**

Relaxin levels in seminal plasma have been associated with positive effects on sperm motility and quality, and thus having potential roles in male fertility. However, the origin of seminal relaxin, within the male reproductive tract, and the moment of its release in the vicinity of spermatozoa remain unclear. Here, we assessed the longitudinal distribution of relaxin and its receptors RXFP1 and RXFP2 in the reproductive tract, sex accessory glands, and spermatozoa of adult boars.

**Methods:**

Spermatozoa were harvested from three fertile boars and reproductive tract (testes and epididymis) and sex accessory gland (prostate and seminal vesicles) tissues were collected post-mortem from each boar. Epididymis ducts were sectioned into caput, corpus, and cauda regions, and spermatozoa were mechanically collected. All samples were subjected to immunofluorescence and/or western immunoblotting for relaxin, RXFP1, and RXFP2 detection. Immunolabeled-spermatozoa were submitted to flow cytometry analyses and data were statistically analyzed with ANOVA.

**Results:**

Both receptors were detected in all tissues, with a predominance of mature and immature isoforms of RXFP1 and RXFP2, respectively. Relaxin signals were found in the testes, with Leydig cells displaying the highest intensity compared to other testicular cells. The testicular immunofluorescence intensity of relaxin was greater than that of other tissues. Epithelial basal cells exhibited the highest relaxin immunofluorescence intensity within the epididymis and the vas deferens. The luminal immunoreactivity to relaxin was detected in the seminiferous tubule, epididymis, and vas deferens ducts. Epididymal and ejaculated spermatozoa were immunopositive to relaxin, RXFP1, and RXFP2, and epididymal corpus-derived spermatozoa had the highest immunoreactivities across epididymal sections. Both vas deferens-collected and ejaculated spermatozoa displayed comparable, but lowest immunofluorescence signals among groups. The entire sperm length was immunopositive to both relaxin and receptors, with relaxin signal being robust in the acrosome area and RXFP2, homogeneously distributed than RXFP1 on the head of ejaculated spermatozoa.

**Conclusions:**

Immunolocalization indicates that relaxin-receptor complexes may have important roles in boar reproduction and that spermatozoa are already exposed to relaxin upon their production. The findings suggest autocrine and/or paracrine actions of relaxin on spermatozoa, either before or after ejaculation, which have possible roles on the fertilizing potential of spermatozoa.

## Background

Sertoli cells build tight junctions among themselves in the testes to form a blood-testis barrier that maintains an enclosed microenvironment favoring normal sperm production. Upon their release within the seminiferous tubule lumen, newly produced spermatozoa are exposed to various molecules that contribute to the acquisition of their final maturation during the epididymal transit [1, 2]. The epididymis is a long duct consisting of various sections with specific functions, known to secrete and reabsorb a variety of molecules that locally interact with spermatozoa to influence their fertilizing potential [3–6]. Relaxin might be one of these molecules, whose presence in the seminal plasma contributes to the crucial roles of this fluid in mammalian fertilization through beneficial effects on sperm motility [7–10].

Relaxin peptide was discovered in the early part of the twentieth century and is characterized as hormone of pregnancy because of its roles during parturition [11]. The first report on relaxin used pregnant guinea pig serum relaxin to demonstrate its beneficial effect on widening the birth canal of non-pregnant animals [12]. Further works later confirmed its positive effects during pregnancy and parturition in various species such as monkeys, pigs, and rodents [13–15]. At present, numerous studies have detected the presence of relaxin in both female (i.e., uterus, ovary) and male (i.e., testes, seminal vesicles, prostate) reproductive tissues, and its functions have been reported in various reproductive and non-reproductive (i.e., brain, pancreas, and kidney) tissues [11].

The tissular distribution of relaxin varies across species and its pleiotropic biological effects are exerted through membrane receptors, known as relaxin family peptide receptor 1 (RXFP1) and 2 (RXFP2) and corresponding to the former leucine-rich repeat-containing G-protein-coupled receptor 7 (LGR7) and 8 (LGR8), respectively [16]. The cellular and physiological effects of relaxin-receptor interactions are better characterized in females, mainly during early and late pregnancy [13, 17, 14, 18], and the co-localization of both relaxin and its receptors in various tissues (i.e., oocytes, cervix, uterus, mammary gland) indicate the existence of possible autocrine and paracrine actions of relaxin [19, 20, 11, 21]. Yet, the presence of both relaxin receptors mRNA and proteins in male reproductive organs is still controversial among studies and species, and consequently, little remains known about relaxin’s presence and roles in male reproductive organs; especially its contribution to the fertilizing potential of spermatozoa [21].

Relaxin is found in male reproductive tissues and accessory glands of various species, and its main production sites appear species-specific [21, 11], with testes, seminal vesicles, and prostate being reported as the major sources of production in various species [21, 22]. Although there are still controversies about the major site of relaxin production in boars [23, 24, 22, 25, 26], a recent study tends to support the testes as the major site of relaxin production, as both RNA transcript and protein were detected in Leydig cells during porcine post-natal development [24]. Nonetheless, the potential roles of testicular relaxin in male reproduction are still controversial, despite various knock-out studies conducted in rodents [21, 27].

Available data in the literature body imply that spermatozoa may be exposed to relaxin within the reproductive tract. The confirmation of this assumption in pigs will contribute to understanding the physiological effects of testicular relaxin on male fertility, as its levels in semen ejaculates appear correlated to sperm motility [8]. Additionally, *in vitro* studies revealed positive effects of relaxin usually extracted from female tissues (e.g., ovary) on post-mating events such as, cervical mucus penetration [28], acrosome reaction, mitochondrial potential, hyperactivation of spermatozoa [29, 30], and oocyte maturation [31]. Hence, it becomes reasonable to question whether it is the male- and/or the female-produced relaxin that provide such effects in the physiological conditions.

From the available literature, it appears that the dynamic expression of relaxin and its receptors throughout the boar reproductive tract, which will provide additional insights into the reproductive impacts of relaxin on male fertility, have not been the focus of previous studies. The current study was undertaken to investigate the main source (s) of relaxin accumulation and the presence of its receptors RXFP1 and RXFP2 in boar reproductive tract (testis, epididymis, and vas deferens) and sex accessory glands (prostate and seminal vesicles). In addition, we profiled the presence of both relaxin and receptor proteins in porcine epididymal and ejaculated spermatozoa. The major findings are that: 1) relaxin and its receptors are present in both reproductive tract and accessory glands, 2) relaxin mainly accumulates within the Leydig cells of the testis, and lower levels were detected in the prostate and seminal vesicles, 3) relaxin is found within the lumen and epithelia of seminiferous tubules and epididymis, in the vicinity of produced and maturing spermatozoa, 4) spermatozoa possess both relaxin and receptor proteins with the amount varying significantly during the epididymal transit, and 5) ejaculated spermatozoa contain both relaxin and receptors RXFP1 and RXFP2 that likely support the long-term roles of male-born relaxin within the female genital tract, through possible autocrine and/or paracrine action.

## Methods

### Chemicals and media

Unless otherwise indicated, all chemicals and reagents were purchased from Sigma-Aldrich (Saint Louis, MI) for general purpose and from Santa Cruz Biotechnologies, Inc. (Santa Cruz, CA) for antibodies.

### Sample preparation

#### Animals and fresh semen collection

Three fertile cross-breed boars of approximately 2.7 ± 0.06 (mean ± sem) years old were used in this study. Fresh semen from each boar was harvested using standard protocol by technicians of a commercial boar stud (Prestage Farms, West Point, MS) and diluted in the Beltsville Thawing Solution (BTS; Minitube of America, Verona, WI). Semen doses were prepared and shipped to our laboratory for experiments. Approximately four hours post-semen collection, all three boars were killed at a local abattoir and reproductive tracts and sex accessory glands (prostate and seminal vesicles) were collected and immediately transported on ice to our laboratory for tissue collections. Ovarian corpus lutea were collected from post-mortem sows at the abattoir for validation studies.

#### Tissue and spermatozoa collections

Reproductive tracts of all boars (n = 3) were dissected into testes, epididymis (caput or head, corpus or body, and cauda or tail), vas deferens, and accessory glands (prostate and seminal vesicles). All tissue samples were recovered and kept on ice. Spermatozoa within each section of the epididymis and vas deferens were mechanically collected (by aspiration with syringes, flushing with a pre-warmed PBS-PVP, and squeezing) and transferred into petri-dishes containing pre-warmed PBS-PVP. Ejaculated and non-ejaculated spermatozoa collected above were subjected to a purification procedure using a single layer percoll gradient (PorciPure, Nidacon: Mölndal, Sweden), as previously described [32]. After centrifugation (600 g - 30 minutes), pelleted spermatozoa devoid of any contaminations (cell debris, extender components, and somatic cells), were washed twice with a cold PBS-PVP through centrifugation (250 g – 5 minutes each). Purified spermatozoa were aliquoted and stored at −20 °C until use for protein analyses. In parallel, subsets of dissected tissues (testis, epididymal caput, corpus and cauda, vas deferens, prostate, and seminal vesicles) were stored at room temperature in containers filled with 10 % formalin for immunofluorescence, while the other subsets were wrapped in aluminum foils, snap-frozen, and stored at −20 °C for protein analyses.

### Western immunoblotting

All samples were thawed at room temperature and total proteins were extracted using complete RIPA buffer containing a protease inhibitor cocktail (Santa Cruz Biotechnologies; Santa Cruz, CA). Total extracted protein were quantified using the Pierce BCA kit (Thermo Fisher Scientific; Rockford, IL) and equivalents of 20 μg were resolved onto 4–12.5 % SDS-PAGE NuPage gels and transferred to PVDF membranes (Millipore Corp, Belford, USA). Similar amounts of ovarian corpus luteum protein extracts were also loaded into the gels for analyses. All membranes were incubated with 500× diluted anti-human relaxin (sc-20652), RXFP1 (sc-50328), or RXFP2 (sc-50327) antibodies, which immunogenicities with pig tissues were previously tested [19]. The immunodetection of proteins was revealed by using the Novex® HRP Chromogenic Western Blot Immunodetection kit (Life Technologies; Grand Island, NY). In parallel, gel electrophoreses loaded with more protein samples (40–60 μg/well) were ran together with the MagicMark™ XP Western Protein Standard (Life Technologies; 10 μl/well) for a longer resolution to better determine the protein sizes after immunoblotting.

### Protein immunofluorescence detection

#### In spermatozoa

Purified sperm samples were fixed in 4 % methanol-free paraformaldehyde (30–60 minutes), permeabilized (30 min) in 1 % Triton-×100, and non-specific binding sites were blocked (60 min) in PBS-PVP solution containing 5 % BSA (v/v). Sperm suspensions were incubated overnight (4 °C) with 100× diluted rabbit anti-human relaxin 1 (sc-20652), RXFP1 (sc-50328), and RXFP2 (sc-50327) in the blocking buffer. Then after, spermatozoa were incubated one hour with 200× diluted FITC labeled goat anti-rabbit secondary antibody in the blocking buffer. Subsets of labeled-spermatozoa were kept in suspensions for a flow cytometry evaluation, while the other subsets were smeared on microscope slides and air-dried under dark. Slides were immediately covered with a DAPI-contained mounting medium to counterstain sperm nuclei for a fluorescence evaluation using a confocal microscope (LSM-510). Samples were washed three times by centrifugation (250 g× 5 min) with PBS-PVP-0.1 % Tween 20 between steps and all procedures were performed at room temperatures otherwise indicated. Samples without any or either primary or secondary antibodies were used as negative controls.

#### In tissues

Tissues were fixed in 10 % formalin at room temperature (30–60 minutes) and blocks were sliced in sections (4–6 μm) and placed on histological slides. Tissue sections were deparafinized, submitted to antigen retrieval (microwave), washed with PBS-PVP and submitted to immediate standard *in situ* immunofluorescence detection of relaxin, RXFP1, and RXFP2 proteins. Additionally, pig ovary sections were processed with the anti-synthetic human RXFP1 antibody (AP23448SU-S; ACRIS Antibodies Inc., San Diego, CA) used at 1/4000 dilution for immunofluorescence signal comparisons with the Santa Cruz anti-human RXFP1.

Reagents used and all procedures were performed as described above for spermatozoa, and samples on the microscope slides were covered with a DAPI-contained mounting medium to counterstain nuclei. The immunofluorescence detection was assessed under a confocal microscope (LSM 510). Samples incubated without primary or secondary antibodies were used as negative controls.

### Flow cytometry evaluation

Suspensions of spermatozoa labeled with relaxin, RXFP1, and RXFP2 antibodies were diluted to approximately 10^6^ cells in 0.8 ml PVP-PVP (1 mg/ml). Samples were analyzed on a Becton Dickinson FACS Aria II flow cytometer (BD Biosciences; San Jose, CA) by excitation with a blue laser (488 nm). The emission signal was measured in FL2 channel centered at 585/42 nm and sorting was performed with a total of 10,000 events analyzed per sample. Data were acquired with the BD FACSDiva™ Version 6.1.3 software and images were treated with the FlowJo software (FlowJo, LLC; Ashland, OR). Three independent experiments were performed and data were expressed as means ± sem. The mean fluorescence of stained cells with both primary and secondary antibodies minus the mean fluorescence of stained cells with only the secondary antibody was used to calculate the percent of labeled cells.

### Confocal microscope imaging

Immediately after immunolabeling, slides containing specimens (spermatozoa and tissue sections) were submitted to fluorescence signal visualization using a Zeiss Laser Scanning Microscope System (LSM 510; Carl Zeiss MicroImaging GmbH, Jena, Germany) with a 488 nm excitation. A (DAPI/Fluorescein/Transmission) filter set was used in single channel mode imaging. Excitation wavelengths of 405 nm/488 nm, Band Pass Emission wavelengths of 420–480 nm (Blue), and Long Pass wavelengths of 505 nm (Green) were acquired at 1024× 1024 pixel formats for imaging purposes.

### Statistical analyses

Only flow cytometry data were statistically analyzed by ANOVA using the General Linear Model procedures of SPSS, version 22 (IBM statistic package package, version 22, Armonk, NY). The homogeneity of variances was performed using the Levene’s test and boars were considered as the random event to evaluate the effect of epididymal section on the immunofluorescence intensity of spermatozoa. The Fisher’s Least Square Difference (LSD) test was performed for pairwise comparisons between protein targets (relaxin, RFXP1, or RXFP2), within and between groups (caput, corpus, cauda, and ejaculated sperm). The threshold of statistical significance was fixed at *p ≤ 0.05*. Data are mean ± sem.

## Results

### Validation of antibodies

**Fig. 1:**
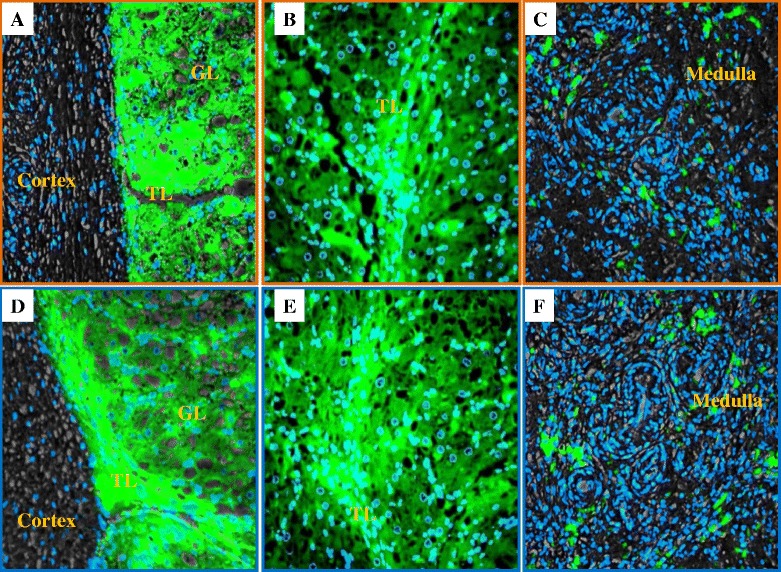
Validation of specificity and immunogenicity of anti-human relaxin antibody on porcine ovarian tissues. Relaxin is detected (Green-FITC) in sections of superficial (**a**/**d**) and deep (**b**/**e**) areas of the corpus luteum, as well as the medulla sections of the sow ovary. Micrographs **a**, **b** and **c** correspond to the labeling with commercial antibody (Santa Cruz Inc., Biotechnology) and micrographs **d**, **c**, and **f** with the homemade porcine antibody (gift from Dr. Bagnell). GL = Granulosa lutein cells, TL = Theca lutein cells, Magnification = 200×. Nuclei are counterstained in blue with DAPI

**Fig. 2:**
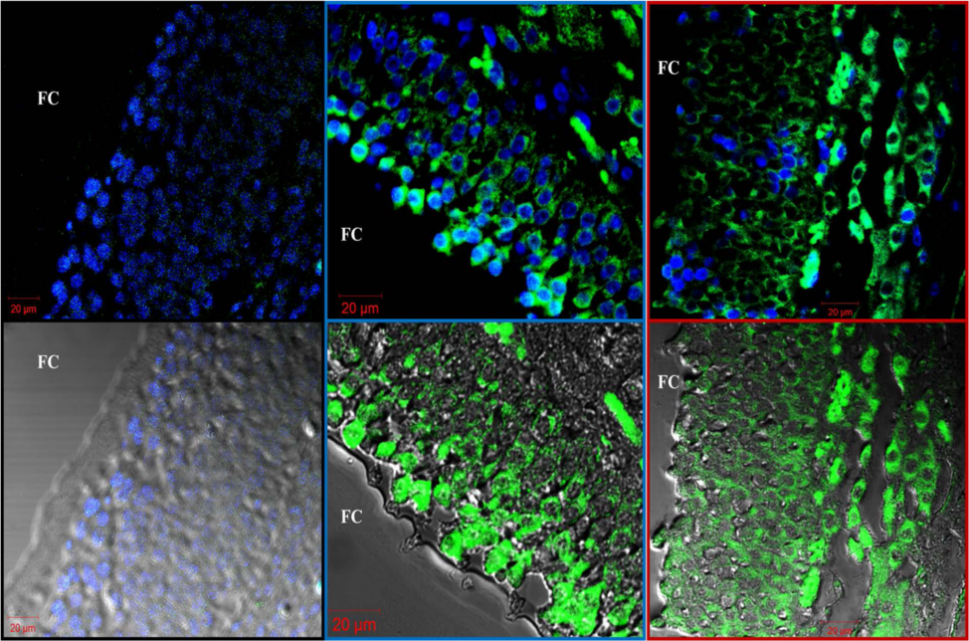
Immuno-fluorescence comparison of two commercial anti-human RXFP1 antibodies on porcine ovarian tissues. Micrograph show control (no primary antibody; black frame), ACRIS (blue frame), and Santa Cruz (red frame) antibodies. Relaxin receptor RXFP1 is generally stained in Green-FITC and mostly seen in the superficial or plasma membrane regions of (mural granulosa) cells lining the internal follicle wall. Upper panel micrographs indicate cells counterstained with DAPI for the nuclei visualization, while the lower panel indicate overlays imaging with visible and fluorescence lights. FC = follicular cavity or antrum

**Fig. 3:**
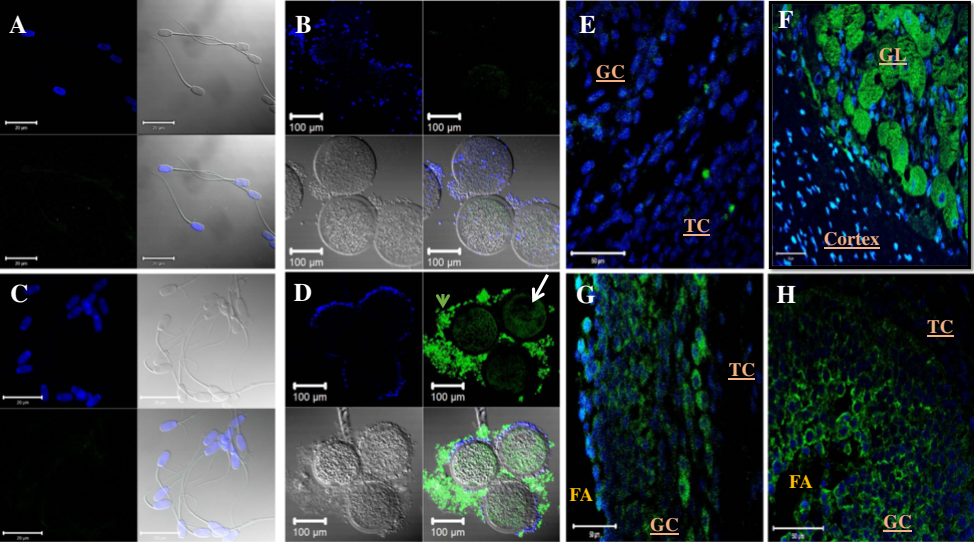
Immunofluorescence of control samples for relaxin, RXFP1, and RXFP2 detection. Images correspond to negative controls, without the primary antibody in sperm (**a**), cumulus-oocyte complex (**b**) and ovary section (**e**) preparations. Absence or weaker green FITC fluorescence signals were observed following sample incubations with FITC-conjugated antibody only. An illustration is shown in micrograph **c**. Cumulus-oocyte complexes incubated with anti-relaxin are shown in micrograph **d**. Micrographs **f**, **g**, and **h** are ovary sections incubated with anti-relaxin, anti-RXFP1, and anti-RXFP2 antibodies, respectively. Cells nuclei are counterstained in blue with DAPI, while protein of interests appeared green (FITC). Micrographs **a**, **b**, **c**, and **d** show stained nuclei, bright-light, green FITC-fluorescence, and the combination of all. Arrow head and arrow respectively indicate the cumulus cells and the oocyte. GC = Granulosa cells; GL = Granulosa Lutein cells; TC = Theca cells; FA = follicle antrum

**Fig. 4:**
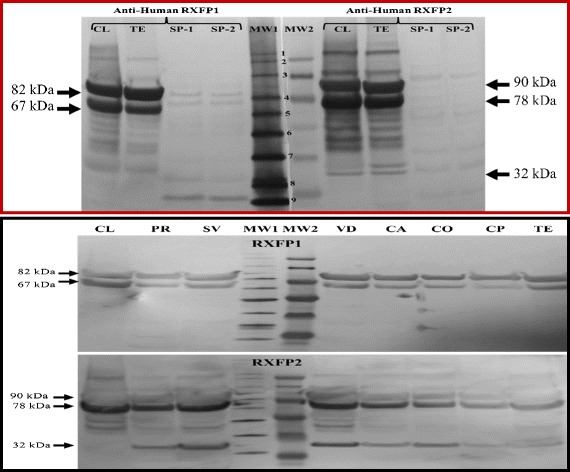
Representative RXFP1 and RXFP2 western immunoblotting gels. Equal amounts (20–60 μg) of total proteins obtained from various pig tissues were resolved on 4–12.5 % SDS-PAGE gels, transferred to PVDF membranes, and immunoblotted with commercial anti-RXFP1 (sc-50328) and anti-RXFP2 (sc-50327) antibodies. Proteins were extracted from prostate (PR), seminal vesicles (SV), vas deferens (VD), and epididymis sections of cauda (CA), corpus (CO), caput (CP), and testicular homogenate (TE). Total protein extracts of sow ovarian corpus lutea (CL) were used as the positive control. Immunodetected proteins were revealed using a colorimetric technique (Novex® HRP Chromogenic). MW1 and MW2 correspond to two different molecular markers, MagicMark™ XP Western Protein Standard (1 = 220 kDa; 2 = 120 kDa; 3 = 100 kDa; 4 = 80 kDa; 5 = 60 kDa; 6 = 50 kDa; 7 = 40 kDa; 8 = 30 kDa; 9 = 20 kDa

Ovarian corpus lutea of cyclic sows were used for validation studies due to their known expression of both relaxin and its receptors [13, 11]. For relaxin detection, we compared the immunofluorescence of a commercial rabbit anti-human relaxin and RXFP1 with a home-made rabbit anti-pig relaxin (gift from Dr. Carol Bagnell at Rutgers University, NJ, USA; Fig. 1) and another commercial source of anti-RXFP1 (ACRIS) reported to be immunoreactive to pig tissues. Antibodies revealed comparable immunolocalization and immunofluorescence intensities in the ovarian corpus luteum and medulla, for anti-relaxin and mural granulosa cells, for anti-RXFP1 (Figs. 1 and 2). Figure 3 shows immunofluorescence detection of relaxin, RXFP1, and RXFP2 in pig samples using commercial antibodies. Weaker or no FITC fluorescence signals were observed in the negative controls, constituted of fixed spermatozoa (micrographs A, B, and C) and sow ovary sections (E). However, the presence of relaxin was detected in granulosa lutein cells of the corpus luteum (micrograph F) and cumulus cells, a part of the mural granulosa cells layer that surrounding the oocyte (micrograph). The presence of RXFP1 and RXFP2 was also detected in the mural granulosa cells (micrographs G and H, respectively). Furthermore, the validation of anti-human RXFP1 and RXFP2 after longer electrophoresis and use of appropriate protein marker standard (MagicMark™ XP) allowed the confirmation of the reported sizes of RXFP1 (~82 and 67 kDa isoforms) and RXFP2 (~ 90 and 78 kDa isoforms) (Fig. 4). Both receptor sizes remained unchanged between all samples (corpus luteum or CL. Testes or TE, ejaculated spermatozoa at high – SP-1 - or low – SP-2 - concentrations) and displayed similar band sizes, while the RXFP2 band appeared lower than RXFP1 in spermatozoa (Fig. 4).

### Western immunoblotting

All tissue samples (testes, prostate, seminal vesicles, vas deferens, and epididymal caput, corpus and cauda) revealed band sizes that were comparable to those detected in the corpus luteum, and corresponding to RXFP 1 and RXFP 2 receptors (Fig. 4). An additional protein band of approximately 32 kDa was detected with the anti-RXFP2 antibody in male tissues exclusively. The bands of 82 kDa (for RXFP 1) and 78 and 32 kDa (for RXFP2) were strongly detected across tissue samples.

### Tissular immunofluorescence detection of relaxin

**Fig. 5:**
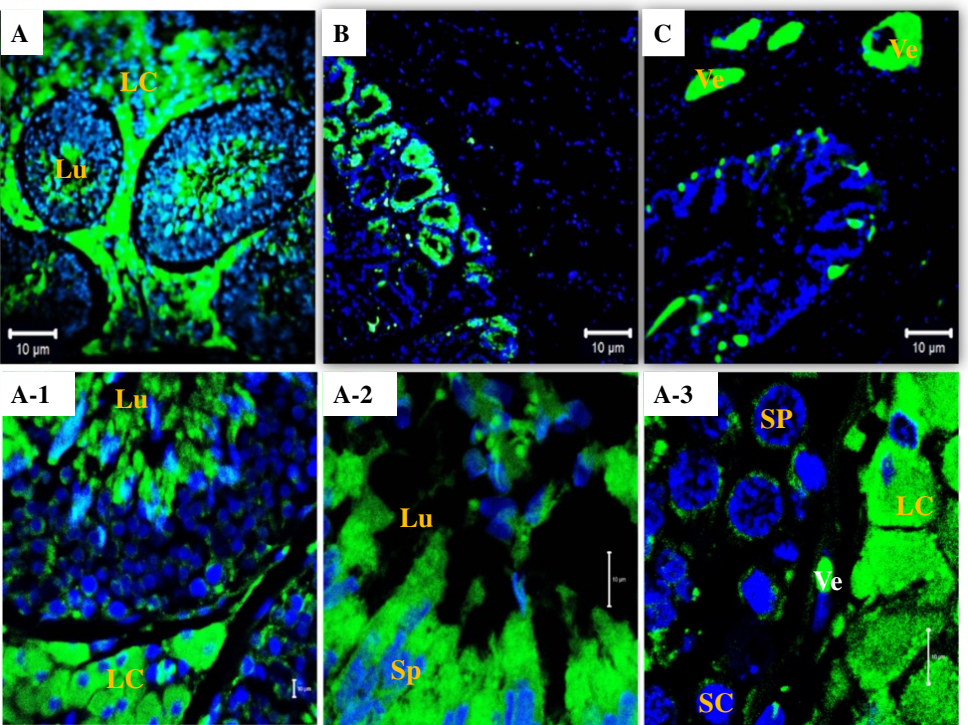
Immunodetection of relaxin in boar reproductive tract and accessory glands. Relaxin signal (green fluorescence) is detected in boar testes (**a**), prostatic glands (**b**), and seminal vesicles (**c**). In the testis, relaxin is detected in interstitial or Leydig cells (LC) and lumen (Lu) of seminiferous tubules (in A-1) and vicinity of spermatids or Sp (in A-2), in spermatocytes (SP) and Sertoli cells or SC (in A-3), and blood vessels (Ve). Scale bars = 10 μm

**Fig. 6:**
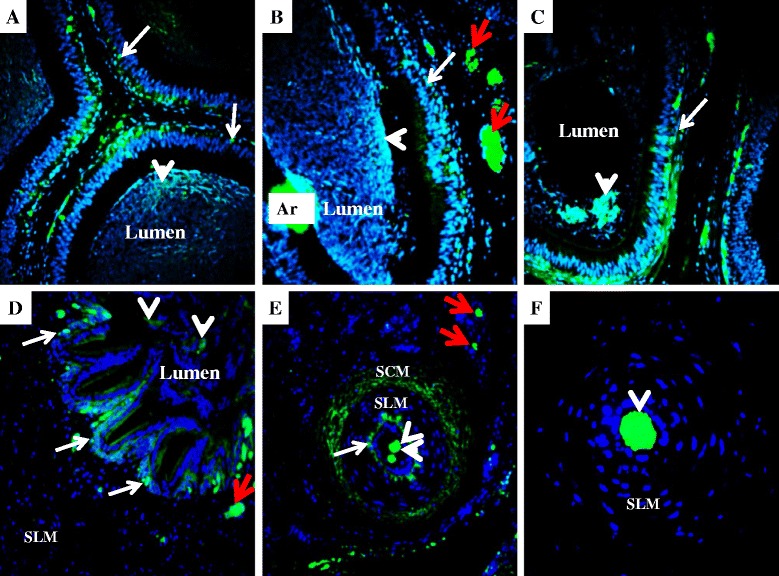
Detection of relaxin in boar epididymis and vas deferens. Relaxin (green fluorescence) is detected in the epididymal caput (**a**), corpus (**b**), and cauda (**c**). Detection is also found in the proximal (**d**), middle (**e**), and distal (**f**) sections of the vas deferens. Signals are usually found in the basal cells (white arrows) and lumen of all epididymal and vas deferens sections, in the vicinity of spermatozoa (white arrow heads). Red arrows indicate relaxin signals found in the blood vessels. Nuclei are counter-stained in blue. Ar = Fluorescence artefact area; SLM = Smooth Longitudinal Muscle; SCM = Smooth Circular Muscle; 200× magnification

Higher relaxin detection signal was found in the testis tissue compared to the prostate and seminal vesicle tissues (Fig. 5A, B, & C). The testicular signal was mainly seen in the interstitium (Leydig cells) and additional immunoreactivities were found in the lumen of seminiferous tubules, spermatids (Fig. 5A-1 & A-2), Sertoli cells, and spermatocytes (Fig. 5A-3). In the epididymis, relaxin signals were pronounced in epithelial principal and epithelial basal cells (Fig. 6A-D). In the middle section of the vas deferens, the smooth circular muscle (SCM) layer showed immunopositive reactivity to relaxin, while the longitudinal (SLM) layer appeared immunonegative (Fig. 6E/F). Figure 6 (A to F) shows relaxin immunoreactivity throughout the epididymis and vas deferens lumen, in the vicinity of the luminal spermatozoa. Finally, the lumen of blood vessels within the reproductive tract and accessory glands appeared immunopositive to relaxin (Figs. 5C/A-3 and 6B-E).

### Sperm immunofluorescence and flow cytometry evaluation

**Fig. 7:**
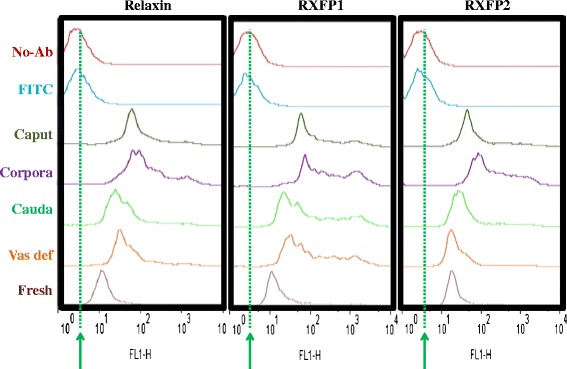
Graphical representations of relaxin, RXFP1, and RXFP2. The figure represents FlowJo graphical layouts of flow cytometry analyses, following immunostaining of ejaculated (freshly collected), epididymal (caput, corpus, and cauda), and vas deferens spermatozoa. Controls corresponded to spermatozoa incubated without any (No-Ab) or only FITC-conjugated secondary (FITC) antibodies. Signal detection peaks of both controls, in each targeted protein, were comparable at the lowest levels (Green lines and arrows) in comparison to other groups

**Fig. 8:**
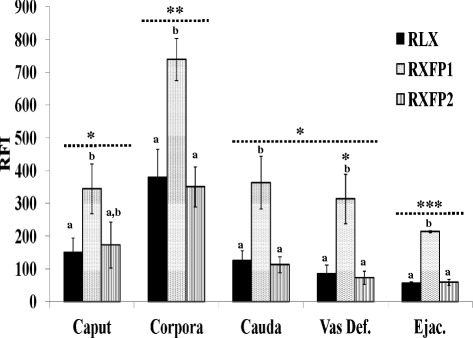
Mean fluorescence detection of relaxin, RXFP1, and RXFP2 in epididymal and freshly ejaculated boar spermatozoa using flow cytometry. ^*,**,***^ For each protein target (relaxin, RXFP1, and RXFP2), asterisks indicate significant differences between groups (caput, corpus, cauda, and ejaculated). Letters (**a**, **b**, **c**) show significant differences between the protein targets within the same group (P < 0.05; ANOVA-1). Data are means ± sem of 3 boars

Flow cytometry analyses revealed that the majority of spermatozoa were successfully labeled with anti-human relaxin (80 ± 6 % to 93 ± 1 %), RXFP1 (86 ± 5 % to 94 ± 1 %), and RXFP2 (79 ± 10 % to 95 ± 1 %) antibodies. The graphical representations of antibody labeling in Fig. 7 indicate the lowest fluorescence or background signals in control groups, consisting of spermatozoa with no antibody (No-Ab) or only the secondary antibody (FITC). Fluorescence intensities of spermatozoa labeled with anti-relaxin, RXFP1, or RXFP2 antibodies were markedly greater than those of the control groups, as depicted by the right-shifted fluorescent intensity curves within the same protein target (Fig. 7). With the control groups as references, ejaculated spermatozoa consistently exhibited the narrowest fluorescence signal shifts (green arrows in Fig. 7), regardless of the protein targets (relaxin, RXFP1, and RXFP2), which coincided with their lowest mean fluorescence intensities compared to other groups (Caput, Corpus, Cauda and Vas deferens - Fig. 8: ^***^P < 0.05; ANOVA-1). Flow cytometry data show that corpus-derived spermatozoa had significantly higher mean fluorescence intensities than any other groups, irrespective of the protein target (Fig. 8: ^*^P < 0.05; ANOVA-1). Within-group comparisons showed that RXFP1 mean fluorescence signals were significantly higher than those of relaxin and RXFP2, in all groups (Fig. 8: ^a, b, c^P < 0.05; ANOVA-1), except in the caput. Both relaxin and RXFP2 signals remained comparable within each group (P > 0.5; Fig. 8), and the expression patterns of both receptors mirrored relaxin detection throughout the epididymis.

### Immunofluorescence detection of relaxin, RXFP1, and RXFP2 in spermatozoa

**Fig. 9:**
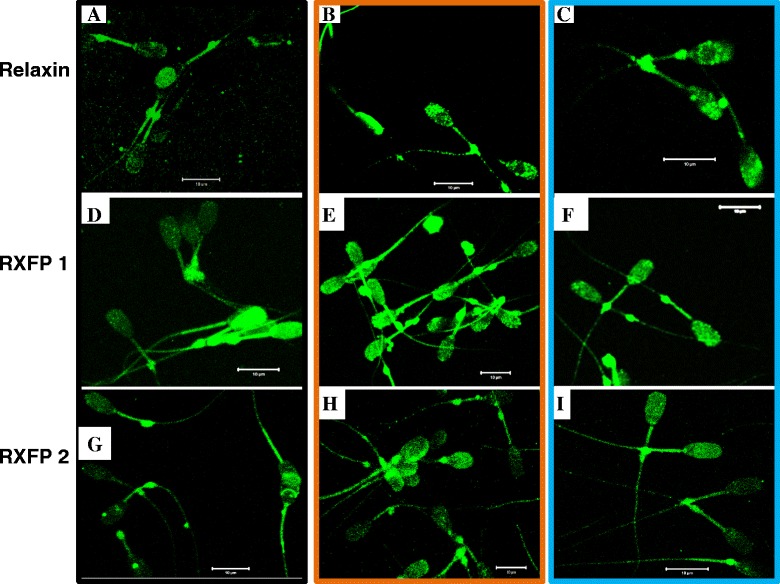
Detection of relaxin, RXFP1, and RXFP2 in epididymal boar spermatozoa. Spermatozoa were collected from epididymal caput (**a**, **d**, and **g**), corpus (**b**, **e**, and **h**), and cauda (**c**, **f**, and **i**) of boars, for the immunodetection of relaxin (**a**, **b**, and **c**), RXFP1 (**d**, **e**, and **f**), and RXFP2 (**g**, **h**, and **i**). Scale bars = 10 μm

**Fig. 10:**
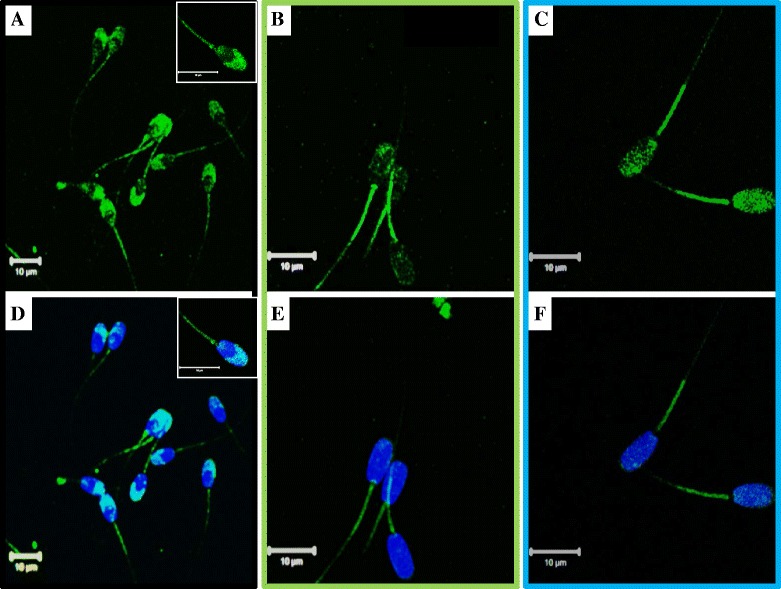
Detection of relaxin, RXFP1, and RXFP2 in ejaculated boar spermatozoa. The green-FITC fluorescence indicates relaxin (**a/d**), RXFP1 (**b/e**), and RXFP2 (**c/f**) detection. Corresponding micrographs **d**, **e**, and **f** show the blue nuclei counterstained with DAPI. Inserts in micrographs **a** and **d** are zooms of the sperm head and mid-piece

Representative images of the immunodetection of relaxin, RXFP1, and RXFP2 in epididymal and ejaculated spermatozoa are shown in Figs. 9 and 10, respectively. There was a great heterogeneity of sperm labeling with each antibody, and all targeted proteins were detected on the entire length (head, mid-piece, and tail) of both epididymal (Fig. 9) and ejaculated (Fig. 10) spermatozoa. However, the relaxin signal appeared more localized in the acrosome area and RXFP2 signals were more homogenous than RXFP1 in ejaculated sperm heads.

## Discussion

Decades after its discovery [12], relaxin was and still is considered as a hormone of pregnancy and parturition despite its detection in various males species, including pigs. At present, numerous studies in rodents have brought strong evidences indicating the crucial but reversible roles of relaxin on male reproduction [33–35]. Here, we show that (i) boar reproductive tract and sex glands express relaxin receptors, (ii) boar testes rather than prostate and seminal plasma accumulate higher levels of relaxin, (iii) relaxin protein is present in the vicinity of spermatozoa upon the onset of spermatogenesis, (iv) spermatozoa possess relaxin and its receptors, suggesting autocrine and/or paracrine actions during their epididymal and post-epididymal lifespan.

### Validation of relaxin, RXFP1, and RXFP2 antibodies

High levels of relaxin are detected in ovarian corpus lutea, which are known as the major source of relaxin production during pregnancy in pigs [13]. Thus, we used ovarian tissues to test the immunogenicity and specificity of a commercial rabbit antibody that targets the full length of human relaxin 1 to detect relaxin in boar tissues. This antibody has been used in previous studies to target relaxin in rat corpus luteum [36] and pig corpus luteum, germ cells, and embryos [19]. The immunofluorescence localization obtained with the commercial relaxin mirrored the rabbit anti-porcine relaxin serum signals, with various sections of the ovary stained alike. In previous studies, both antibodies successfully targeted relaxin in corpus luteum (granulosa and theca lutein) cells of various species [23, 13, 25, 37–40].

As to relaxin receptors, we used anti-human RXFP1 and RXFP2 antibodies because of the high degree of sequence conservations among these receptors across species. Indeed, the immunogenic sequences of each receptor have been shown to share over 99 % similarity across various mammals (e.g., cattle, chimpanzee, dog, horse, human, and mice) [19]. The successful labeling of over 80 % of spermatozoa in the current study further confirms the high immunogenicity of the used commercial antibodies. Moreover, the profile of bands detected with both anti-human RXFP1 and RXFP2 antibodies were similar between the male tissues and the corpus luteum, used as positive control [41]. Most importantly, the comparison of two different sources of anti-human RXFP1 antibodies (Santa Cruz vs. ACRIS antibody) showed comparable immunohistofluorescence of porcine ovary sections. The ACRIS antibody is reported to interact with porcine RXFP1 protein [42]. The current study confirms the estimated size of 82 kDa as the mature isoform of porcine RXFP1 using the anti-human antibody from Santa Cruz Inc., Biotech, while the technical improvements (better protein resolution and appropriate molecular protein weight markers) allowed for accurate size estimations of the mature and (90 kDa) immature (78 kDa) isoforms of RXFP2, which reverse our previous report [19] and corroborate with the commonly reported size of the mature RXFP2 isoform.

### Tissular immunofluorescence detection of relaxin

The intense immunoreactivity of relaxin within the testicular interstitium (Leydig cells) was consistent with previous reports in boars employing the immunological approach [23, 43, 24]. Kato et al., used up to 10 month old pigs to detect relaxin protein signals that were restricted to the Leydig cells [24], and therefore confirmed a pioneer study that reported weaker relaxin signals in boar Sertoli cells [43]. Compared to previous studies in boars [43, 24, 25, 23], we used a purified commercial antibody to confirm the presence of relaxin in various areas of the boar reproductive tract (i.e., Sertoli and seminiferous epithelial cells, spermatocytes, and lumen of seminiferous tubules and epididymis) that were still controversial in previous reports [21, 26]. Our findings provide a strong evidence of boar spermatozoa being exposed to testicular relaxin upon their release in the seminiferous lumen.

Although its main source of production in boars remains unclear [26], the combination of our findings, indicating differential immunofluorescence intensities of relaxin between tissues, with a recent report, showing higher expression levels of relaxin mRNA in boar testes [24] establishes strong evidences of the testes being the major production and accumulation sites of relaxin in boars. Nonetheless, the possibility of testis-produced relaxin being routed out for final processing and/or further accumulation in the prostate and seminal vesicles cannot be ruled out. A previous study has reported that boar testes mainly accumulate immature pro-pre- and pro-relaxin, due to the absence of the necessary cleavage enzyme [24], which may be an evolutionary adaptation of mammals to the lower temperature of scrotal testes. Available reports suggest a possible shift of bio-active relaxin production from abdominal testes in animals such as birds [44] and dogfish sharks [45] to sex accessory glands in mammals [21].

Furthermore, the current study indicates a greater immunodetection of relaxin in prostatic glands than that seen in seminal vesicles, which may contradict a previous suggestion that seminal vesicles produce more relaxin than prostate in boars [22], unless the larger size of the seminal vesicle is being considered for this production.

In addition, the robust immunoreactivity of relaxin within the local blood vessels is in agreement with a previous report in boars [7], and may likely be indicative of the possible back and forth vascular transportation of both immature and mature relaxin occurring between the testis and accessory glands. With the antibody used in this study targeting both immature and mature relaxin, it could then be speculated that testicular-born (immature) relaxin may gain maturity within the accessory glands [24], before being redirected via the blood vessels to direct effects during testicular growth [34], spermatogenesis [46, 24], and sperm maturation. Accessory glands are also known to dump their secretions that would also contain relaxin into the post-vas deferens duct to constitute the seminal plasma of the ejaculate and having direct effects on sperm functions (i.e., motility).

### Tissular detection of relaxin receptors

All analyzed tissues (epididymal caput, corpus, and cauda, vas deferens, prostate, and seminal vesicles) showed various RXFP1 and RXFP2 band sizes that were comparable to those found in previous studies, using porcine granulosa cells, germ cells, and corpus luteum [19, 24] and other *in vitro* models [47, 48]. At this point, it is important to mention that RXFP2 bands were comparable although their size values were wrongly reported (78 and 62 kDa in our previous study, instead of 90 and 78 kDa). These receptors have been detected at either or both mRNA and protein levels in male reproductive tissues of various species [21]. As suggested by previous authors [49, 50], the existence of multiple receptor isoforms that are likely products of RNA splicing variants may correspond to mature (82 kDa and 90 kDa) and immature (67 kDa and 78 kDa) isoforms of RXFP1 and RXFP2, respectively. Although the 32 kDa RXFP2 band could be a truncated extracellular isoform the detected immature and mature bands appeared at slightly smaller sizes compared to their human and rat counterparts, which may be due to differential glycosylation levels between species [51, 52, 49, 50, 47]. Because these bands may correspond to isoforms synthesized for cell membrane delivery (mature), intracellular storage (immature), and extracellular excretion (truncated), the differential expression profiles of RXFP1 and RXFP2 on western immunoblotting may indicate their specific involvement in boar reproduction.

Preponderant physiological roles of relaxin-RXFP1 complexes could be speculated in testicular and epididymal cells given the higher level of immature RXFP2 and in mature spermatozoa, given the higher level of mature RXFP1 This speculation would be in agreement with previous works reporting the presence of relaxin binding sites and RXFP1 mRNA in porcine Leydig and Sertoli cells [24, 25] and expression of RXFP1 in rat Sertoli and post-meiotic germ cells [53], rhesus monkey testis [54], and human spermatozoa [29].

Unlike RXFP1, RXFP2 protein appears mainly in its immature and truncated extracellular forms in all examined tissues. This may be surprising giving the presence and roles of its major ligand, the relaxin-like factor (RFL) or insulin-like factor 3 (INSL3), on boar testes [55]. Nonetheless, the main roles of INSL3 have been described during early development [56, 57], while its protection against pro-apoptotic agents in adult animals [58] may not be preponderant in high fertile adult animals, such as the boars utilized in the current study (2.7 ± 0.06 year old).

Yet, the physiological significance of the truncated extracellular isoform remains unknown and would more likely be non-functional. However, the immature isoforms of both receptors are expected to serve as pools of intracellular sequestrated receptors. This receptors’ retention will allow a rapid mobilization to the plasma membrane, as needed and therefore keeping the cells at a high level of physiological responsiveness [59, 51]. The existence of such receptor variants in boars may create unforeseeable interactions with relaxin, leading to a possible complexity of the relaxin-receptor systems that remain to be elucidated. Nonetheless, the use of knock-out approaches in mice has permitted more insights into the physiological relevance of the relaxin-receptor systems, but there are still controversies in the findings according to factors such as the first generation of transgenic mice models and strains [21, 27]. Consequently, the precise and full roles of relaxin signaling during spermatogenesis and sperm maturation are still unclear. The current study shows the apparent co-detection of relaxin and its receptors in boar reproductive tissues, which indicates possible local effects of relaxin on male fertility.

### Immunofluorescence detection of relaxin and its receptors in spermatozoa

Previous studies have described the presence of RXFP1 [24] and RXFP2 [55] mRNA transcripts in boar germ cells and mature spermatozoa, while the presence of relaxin transcripts in mature spermatozoa were undetected [19]. Here, we reveal the presence of corresponding proteins, as well as relaxin on both epididymal and ejaculated boar spermatozoa. The observed variations in relative expression of these proteins on spermatozoa throughout the epididymal transit can mostly be explained by the active secretory and reabsorption functions of the epididymis [60], as the transcriptional and translational machineries of spermatozoa are quasi-completely reduced.

The epididymal function is likely concomitant with the progressive decrease of sperm binding relaxin and receptors, as well as the increased sperm density to facilitate interactions between luminal fluid contents and spermatozoa achieving their epididymal maturation [1]. Therefore, the intense immunoreactivity of relaxin within the vas deferens lumen supports the possible contribution of testicular- and epididymal-born relaxin to sperm maturation and beyond. It is more likely that this luminal relaxin will later combines with prostatic and seminal vesicular relaxin in the seminal plasma, to provide further effects on sperm function via paracrine actions during its journey towards fertilization [28, 8, 61].

Most importantly, our findings also suggest autocrine actions due to the co-localization of both relaxin and its receptors on mature spermatozoa, and thus contributing to support prolonged roles of male–born relaxin until or beyond fertilization. Indeed, beneficial effects of autocrine actions of relaxin-receptor complexes are expected on sperm motility and interactions with its surrounding within the female genital tract, as relaxin is capable of various actions that include the breakdown of tissular metalloprotein surface matrix [62], that may also affect the sperm-egg interactions and acrosome reaction of spermatozoa [29].

## Conclusions

The current findings (*i*) support the testes being the main site of relaxin production in boar, (*ii*) reveal that spermatozoa are exposed to relaxin upon their release into the seminiferous tubule lumen, and (*iii*) indicate that male-born relaxin and its RXFP1 and RXFP2 receptors found on mature spermatozoa may have important roles, via autocrine and paracrine mechanisms, on sperm motility and viability.

## Abbreviations

RXFP: Relaxin family peptide receptors
ANOVA: Analysis of variances
FITC: Fluorescein isothiocyanate
FACS: Fluorescence-assisted cell sorting
PBS: Phosphate buffered solution
PVP: Polyvinylpyrrolidone
